# Crystal structure of bis­[2-(1*H*-benzimidazol-2-yl)aniline]silver(I) nitrate

**DOI:** 10.1107/S2056989015015315

**Published:** 2015-08-22

**Authors:** Yongtae Kim, Sung Kwon Kang

**Affiliations:** a Department of Chemistry, Chungnam National University, Daejeon 305-764, Republic of Korea

**Keywords:** crystal structure, silver(I) complex, distorted square-planar geometry, benzimidazole, N—H⋯O hydrogen bonds

## Abstract

In the cation of the title salt, [Ag(C_13_H_11_N_3_)_2_]NO_3_, the Ag^I^ atom lies on a crystallographic inversion center and is coordinated by four N atoms from two bidentate 2-(1*H*-benzimidazol-2-yl)aniline ligands in a distorted square-planar geometry. The Ag—N(aniline) bond [2.729 (2) Å] is significantly longer than the Ag—N(imidazole) bond [2.165 (1) Å]. In the ligand, the aniline ring is twisted by 37.87 (6)° from the mean plane of the benzimidazole ring system. The nitrate anion lies on a crystallographic twofold rotation axis which passes through the N atom and one of the O atoms. In the crystal, N—H⋯O hydrogen bonds link the components, forming a layer parallel to the *bc* plane.

## Abstract 2

The geometry around silver(I) metal atom in the title complex is distorted square planar with two normal Ag—N bonds and two long Ag—N bonds.

## Chemical context   

Azole and benzazole derivatives have been of inter­est in an important group in biological systems (Esparza-Ruiz *et al.*, 2011 ▸; Hock *et al.*, 2013 ▸). Benzimidazoles have shown anti­viral and anti­tumor activity (Wang *et al.*, 2007 ▸; Ramla *et al.*, 2007 ▸). Some transition metal complexes with benzimidazole derivatives are important biological mol­ecules (Sánchez-Guadarrama *et al.*, 2009 ▸; Gökçe *et al.*, 2005 ▸). The complexes of silver(I) with a series of benzimidazole-based *N*-heterocyclic carbenes have shown *in vitro* anti­bacterial potential against *E. coli* and *B. subtilis* bacteria (Haque *et al.*, 2015 ▸). Recently, we reported on the synthesis and structural features of a zinc complex with a benzimidazole derivative (Kim & Kang, 2015 ▸). In a continuation of our research in this area, the title complex has been synthesized and characterized by single crystal diffraction.
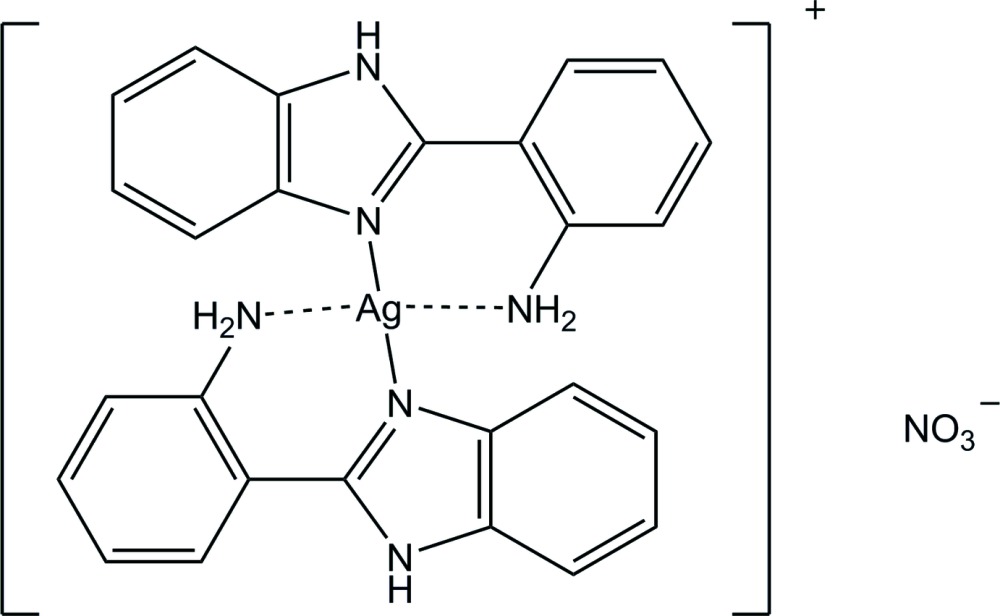


## Structural commentary   

**Figure 1:**
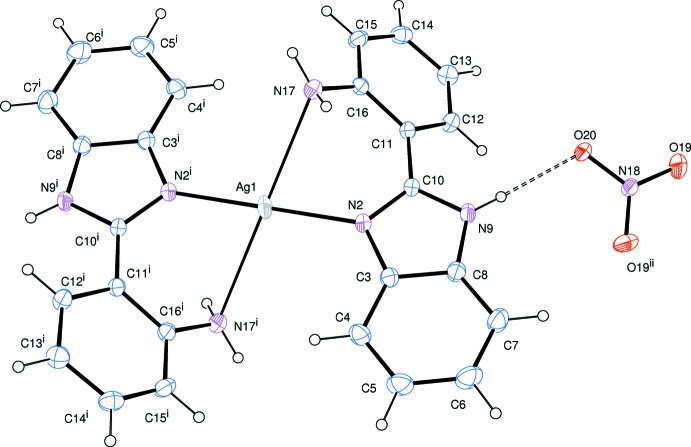
Mol­ecular structure of the title compound, showing the atom-numbering scheme and 30% probability ellipsoids. The N—H⋯O hydrogen bond is indicated by a dashed line. [Symmetry codes: (i) −*x*, −*y*, −*z* + 1; (ii) −*x*, *y*, −*z* + 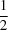.]

The cationic Ag^I^ complex adopts a distorted square-planar geometry with four N atoms of two bidentate 2-(1*H*-benzimidazol-2-yl)aniline ligands (Fig. 1 ▸). The Ag^I^ atom lies on a crystallographic inversion center. The smaller N2—Ag1—N17 angle is 74.8 (1)° and the other is 105.2 (1)°. The benzimidazole ring system (N2–C10) is almost planar with an r.m.s. deviation of 0.015 (2) Å from the corresponding least-squares plane defined by the nine constituent atoms. The dihedral angle between the benzimidazole ring system and the aniline ring is 37.87 (6)°. This twisting is a driving force in the formation of the weak Ag1—N17 bonding in the Ag complex. The Ag1—N17 bond length of 2.729 (2) Å is much longer than the Ag1—N2 bond length of 2.165 (1) Å. Typical Ag—N bond lengths are within the range 2.1–2.4 Å (Gulbransen & Fitchett, 2012 ▸; Pettinari *et al.*, 2013 ▸; Sun *et al.*, 2006 ▸). However, the bond length of 2.729 (2) Å is shorter than the sum of the van der Waals radii of N and Ag atoms (1.55 and 1.70 Å, respectively; Bondi, 1964 ▸). In the heterocyclic imidazole ring, the N2—C10 bond [1.331 (2) Å] is shorter than the other N—C bonds [N2—C3 1.388 (2), C8—N9 1.380 (2), N9—C10 1.352 Å], which means the N2—C10 bond has double-bond character. In the nitrate counter-anion, atoms N18 and O20 lie on a crystallographic twofold rotation axis.

## Supra­molecular features   

**Figure 2:**
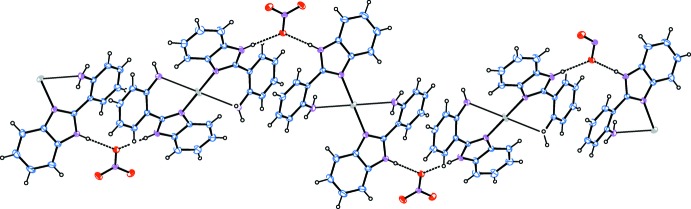
Part of the crystal structure of the title compound, showing mol­ecules linked by inter­molecular N—H⋯O hydrogen bonds (dashed lines).

In the crystal, the N—H group of the 2-(1*H*-benzimidazol-2-yl)aniline ligand inter­acts strongly with the counter-anion, giving rise to a nearly linear hydrogen bond (Table 1 ▸), which forms a zigzag chain along the *c* axis (Fig. 2 ▸). Another weak N—H⋯O hydrogen bond between the NH_2_ group and the anion (Table 1 ▸) links the chains into a layer parallel to the *bc* plane.

## Database survey   

A search of the Cambridge Structural Database (Version 5.36 with one update; Groom & Allen, 2014 ▸) returned 2993 entries for crystal structures of benzimidazoles. Most of them are crystal structures of metal complexes. However, there are only four entries with the ligand 2-(1*H*-benzimidazol-2-yl)aniline or 2-(2-amino­phen­yl)-1*H*-benzimidazole bonded to a transition metal: a Zn complex (Eltayeb *et al.*, 2011 ▸), an Ni (Esparza-Ruiz *et al.*, 2011 ▸), an Re (Machura *et al.*, 2011 ▸) and an Ru (Malecki, 2012 ▸).

## Synthesis and crystallization   

To a stirred solution of Ag(NO_3_) (0.085 g, 0.5 mmol) in aceto­nitrile (5 ml) was added a solution of 2-(1*H*-benzimidazol-2-yl)aniline (0.211 g, 1.0 mmol) in aceto­nitrile (10 ml) at 333 K. After 24 h of stirring, the solution turned ivory in color. Single crystals of the title complex were obtained by slow evaporation of the solvent at room temperature within three weeks.

## Refinement   

Crystal data, data collection and structure refinement details are summarized in Table 2 ▸. H atoms of the NH and NH_2_ groups were located in a difference Fourier map and refined freely [refined distances; N—H = 0.81 (3)–0.89 (3) Å]. Other H atoms were positioned geometrically and refined using a riding model, with C—H = 0.93 Å, and with *U*_iso_(H) = 1.2*U*_eq_(C).

## Supplementary Material

Crystal structure: contains datablock(s) I. DOI: 10.1107/S2056989015015315/is5411sup1.cif

Structure factors: contains datablock(s) I. DOI: 10.1107/S2056989015015315/is5411Isup2.hkl

CCDC reference: 1419095

Additional supporting information:  crystallographic information; 3D view; checkCIF report

## Figures and Tables

**Table 1:** Hydrogen-bond geometry (, )

*D*H*A*	*D*H	H*A*	*D* *A*	*D*H*A*
N9H9O20	0.81(3)	2.05(3)	2.8588(18)	178(3)
N17H17*B*O19^i^	0.89(3)	2.35(3)	3.214(2)	164(2)

Symmetry code: (i) 
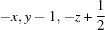
.

**Table 2:** Experimental details

Crystal data	
Chemical formula	[Ag(C_13_H_11_N_3_)_2_]NO_3_
*M*_r_	588.37
Crystal system, space group	Orthorhombic, *P* *b* *c* *n*
Temperature (K)	296
*a*, *b*, *c* ()	11.9903(2), 10.1377(2), 20.1115(5)
*V* (^3^)	2444.63(9)
*Z*	4
Radiation type	Mo *K*
(mm^1^)	0.87
Crystal size (mm)	0.18 0.16 0.15

Data collection	
Diffractometer	Bruker SMART CCD area detector
Absorption correction	Multi-scan (*SADABS*; Bruker, 2002 ▸)
*T*_min_, *T*_max_	0.846, 0.872
No. of measured, independent and observed [*I* > 2(*I*)] reflections	62591, 3043, 2446
*R*_int_	0.034
(sin /)_max_ (^1^)	0.667

Refinement	
*R*[*F* ^2^ > 2(*F* ^2^)], *wR*(*F* ^2^), *S*	0.030, 0.080, 1.06
No. of reflections	3043
No. of parameters	182
H-atom treatment	H atoms treated by a mixture of independent and constrained refinement
_max_, _min_ (e ^3^)	0.44, 0.48

Computer programs: *SMART* and *SAINT* (Bruker, 2002 ▸), *SHELXS2013* (Sheldrick, 2008 ▸), *SHELXL2013* (Sheldrick, 2015 ▸), *ORTEP-3 for Windows* (Farrugia, 2012 ▸) and *publCIF* (Westrip, 2010 ▸).

## supplementary crystallographic information

### Crystal data

**Table:**

[Ag(C_13_H_11_N_3_)_2_](NO_3_)	*D*_x_ = 1.599 Mg m^−^^3^
*M**_r_* = 588.37	Mo *K*α radiation, λ = 0.71073 Å
Orthorhombic, *P**b**c**n*	Cell parameters from 9923 reflections
*a* = 11.9903 (2) Å	θ = 2.6–28.3°
*b* = 10.1377 (2) Å	µ = 0.87 mm^−^^1^
*c* = 20.1115 (5) Å	*T* = 296 K
*V* = 2444.63 (9) Å^3^	Block, colourless
*Z* = 4	0.18 × 0.16 × 0.15 mm
*F*(000) = 1192	

### Data collection

**Table:**

Bruker SMART CCD area-detector diffractometer	2446 reflections with *I* > 2σ(*I*)
Radiation source: fine-focus sealed tube	*R*_int_ = 0.034
φ and ω scans	θ_max_ = 28.3°, θ_min_ = 2.0°
Absorption correction: multi-scan (*SADABS*; Bruker, 2002)	*h* = −15→15
*T*_min_ = 0.846, *T*_max_ = 0.872	*k* = −13→13
62591 measured reflections	*l* = −26→26
3043 independent reflections	

### Refinement

**Table:**

Refinement on *F*^2^	0 restraints
Least-squares matrix: full	Hydrogen site location: mixed
*R*[*F*^2^ > 2σ(*F*^2^)] = 0.030	H atoms treated by a mixture of independent and constrained refinement
*wR*(*F*^2^) = 0.080	*w* = 1/[σ^2^(*F*_o_^2^) + (0.0371*P*)^2^ + 1.1132*P*] where *P* = (*F*_o_^2^ + 2*F*_c_^2^)/3
*S* = 1.06	(Δ/σ)_max_ < 0.001
3043 reflections	Δρ_max_ = 0.44 e Å^−^^3^
182 parameters	Δρ_min_ = −0.48 e Å^−^^3^

### Special details

**Table:**

Geometry. All e.s.d.'s (except the e.s.d. in the dihedral angle between two l.s. planes) are estimated using the full covariance matrix. The cell e.s.d.'s are taken into account individually in the estimation of e.s.d.'s in distances, angles and torsion angles; correlations between e.s.d.'s in cell parameters are only used when they are defined by crystal symmetry. An approximate (isotropic) treatment of cell e.s.d.'s is used for estimating e.s.d.'s involving l.s. planes.

### Fractional atomic coordinates and isotropic or equivalent isotropic displacement parameters (Å^2^)

**Table:**

	*x*	*y*	*z*	*U*_iso_*/*U*_eq_	
Ag1	0.0000	0.0000	0.5000	0.04858 (10)	
N2	−0.04065 (13)	0.18355 (14)	0.45056 (7)	0.0336 (3)	
C3	−0.09293 (15)	0.29248 (18)	0.47845 (9)	0.0340 (4)	
C4	−0.13679 (17)	0.3119 (2)	0.54221 (10)	0.0436 (4)	
H4	−0.1356	0.2450	0.5739	0.052*	
C5	−0.18161 (19)	0.4335 (2)	0.55614 (11)	0.0527 (5)	
H5	−0.2119	0.4487	0.5980	0.063*	
C6	−0.1829 (2)	0.5349 (3)	0.50918 (12)	0.0588 (6)	
H6	−0.2133	0.6161	0.5208	0.071*	
C7	−0.14013 (19)	0.5180 (2)	0.44611 (12)	0.0512 (5)	
H7	−0.1417	0.5852	0.4146	0.061*	
C8	−0.09453 (15)	0.39484 (18)	0.43215 (9)	0.0365 (4)	
N9	−0.04206 (14)	0.34549 (15)	0.37641 (8)	0.0355 (3)	
H9	−0.030 (2)	0.381 (3)	0.3412 (13)	0.056 (7)*	
C10	−0.01033 (14)	0.22026 (16)	0.38965 (8)	0.0300 (3)	
C11	0.05731 (14)	0.14278 (16)	0.34296 (8)	0.0295 (3)	
C12	0.13818 (14)	0.20841 (18)	0.30566 (9)	0.0364 (4)	
H12	0.1448	0.2995	0.3095	0.044*	
C13	0.20848 (15)	0.1415 (2)	0.26327 (9)	0.0405 (4)	
H13	0.2605	0.1871	0.2378	0.049*	
C14	0.20058 (17)	0.00563 (19)	0.25906 (9)	0.0407 (4)	
H14	0.2497	−0.0409	0.2320	0.049*	
C15	0.12026 (16)	−0.06123 (18)	0.29469 (9)	0.0386 (4)	
H15	0.1155	−0.1525	0.2911	0.046*	
C16	0.04616 (16)	0.00555 (16)	0.33594 (9)	0.0320 (4)	
N17	−0.03508 (17)	−0.06622 (18)	0.37016 (8)	0.0410 (4)	
H17A	−0.092 (2)	−0.021 (2)	0.3769 (11)	0.042 (6)*	
H17B	−0.048 (2)	−0.145 (3)	0.3528 (13)	0.068 (8)*	
N18	0.0000	0.5893 (2)	0.2500	0.0314 (4)	
O19	0.02905 (14)	0.64820 (15)	0.19918 (8)	0.0534 (4)	
O20	0.0000	0.4645 (2)	0.2500	0.0446 (5)	

### Atomic displacement parameters (Å^2^)

**Table:**

	*U*^11^	*U*^22^	*U*^33^	*U*^12^	*U*^13^	*U*^23^
Ag1	0.07365 (18)	0.03391 (13)	0.03818 (13)	0.00637 (10)	0.00441 (10)	0.01569 (8)
N2	0.0448 (8)	0.0272 (7)	0.0287 (7)	−0.0002 (6)	0.0025 (6)	0.0041 (6)
C3	0.0372 (9)	0.0314 (9)	0.0332 (8)	−0.0026 (7)	0.0043 (7)	0.0015 (7)
C4	0.0487 (10)	0.0466 (11)	0.0355 (9)	−0.0064 (9)	0.0113 (8)	0.0006 (8)
C5	0.0548 (12)	0.0550 (13)	0.0483 (12)	−0.0016 (10)	0.0205 (10)	−0.0094 (10)
C6	0.0637 (15)	0.0460 (12)	0.0666 (15)	0.0147 (11)	0.0202 (11)	−0.0103 (11)
C7	0.0612 (13)	0.0357 (11)	0.0565 (13)	0.0134 (9)	0.0141 (11)	0.0052 (9)
C8	0.0413 (10)	0.0312 (9)	0.0369 (9)	0.0029 (7)	0.0066 (7)	0.0026 (7)
N9	0.0483 (8)	0.0268 (8)	0.0315 (8)	0.0070 (6)	0.0069 (7)	0.0071 (6)
C10	0.0359 (8)	0.0250 (8)	0.0291 (8)	−0.0005 (6)	0.0001 (6)	0.0034 (6)
C11	0.0344 (8)	0.0267 (8)	0.0273 (8)	0.0027 (7)	−0.0026 (6)	0.0022 (6)
C12	0.0386 (9)	0.0290 (8)	0.0415 (10)	−0.0005 (7)	0.0013 (7)	0.0020 (7)
C13	0.0335 (9)	0.0455 (11)	0.0427 (10)	−0.0002 (8)	0.0043 (7)	−0.0007 (8)
C14	0.0381 (9)	0.0439 (11)	0.0402 (11)	0.0089 (8)	−0.0035 (7)	−0.0098 (8)
C15	0.0467 (10)	0.0280 (9)	0.0411 (10)	0.0051 (8)	−0.0075 (8)	−0.0072 (7)
C16	0.0386 (8)	0.0298 (9)	0.0278 (8)	−0.0011 (7)	−0.0074 (7)	0.0013 (6)
N17	0.0558 (10)	0.0306 (9)	0.0366 (9)	−0.0083 (8)	0.0003 (8)	0.0004 (7)
N18	0.0370 (10)	0.0232 (10)	0.0341 (10)	0.000	−0.0049 (9)	0.000
O19	0.0731 (10)	0.0381 (8)	0.0491 (8)	−0.0022 (7)	0.0064 (7)	0.0155 (7)
O20	0.0759 (14)	0.0209 (8)	0.0368 (10)	0.000	0.0055 (9)	0.000

### Geometric parameters (Å, º)

**Table:**

Ag1—N2^i^	2.1653 (14)	C10—C11	1.468 (2)
Ag1—N2	2.1653 (14)	C11—C12	1.395 (2)
Ag1—N17	2.7288 (17)	C11—C16	1.405 (2)
N2—C10	1.331 (2)	C12—C13	1.377 (3)
N2—C3	1.388 (2)	C12—H12	0.9300
C3—C8	1.394 (2)	C13—C14	1.383 (3)
C3—C4	1.400 (2)	C13—H13	0.9300
C4—C5	1.374 (3)	C14—C15	1.379 (3)
C4—H4	0.9300	C14—H14	0.9300
C5—C6	1.396 (3)	C15—C16	1.391 (3)
C5—H5	0.9300	C15—H15	0.9300
C6—C7	1.379 (3)	C16—N17	1.397 (3)
C6—H6	0.9300	N17—H17A	0.83 (2)
C7—C8	1.392 (3)	N17—H17B	0.89 (3)
C7—H7	0.9300	N18—O19^ii^	1.2340 (17)
C8—N9	1.380 (2)	N18—O19	1.2340 (17)
N9—C10	1.352 (2)	N18—O20	1.265 (3)
N9—H9	0.81 (3)		
			
N2^i^—Ag1—N2	180.0	N9—C10—C11	122.12 (15)
N2^i^—Ag1—N17	105.23 (6)	C12—C11—C16	118.98 (16)
N2—Ag1—N17	74.77 (5)	C12—C11—C10	118.21 (15)
C10—N2—C3	105.83 (14)	C16—C11—C10	122.78 (16)
C10—N2—Ag1	126.97 (12)	C13—C12—C11	121.57 (17)
C3—N2—Ag1	126.86 (11)	C13—C12—H12	119.2
N2—C3—C8	109.18 (15)	C11—C12—H12	119.2
N2—C3—C4	130.65 (17)	C12—C13—C14	119.08 (18)
C8—C3—C4	120.14 (17)	C12—C13—H13	120.5
C5—C4—C3	117.37 (19)	C14—C13—H13	120.5
C5—C4—H4	121.3	C15—C14—C13	120.38 (18)
C3—C4—H4	121.3	C15—C14—H14	119.8
C4—C5—C6	121.8 (2)	C13—C14—H14	119.8
C4—C5—H5	119.1	C14—C15—C16	121.11 (17)
C6—C5—H5	119.1	C14—C15—H15	119.4
C7—C6—C5	121.8 (2)	C16—C15—H15	119.4
C7—C6—H6	119.1	C15—C16—N17	119.05 (17)
C5—C6—H6	119.1	C15—C16—C11	118.75 (17)
C6—C7—C8	116.3 (2)	N17—C16—C11	122.18 (17)
C6—C7—H7	121.8	C16—N17—Ag1	103.64 (11)
C8—C7—H7	121.8	C16—N17—H17A	111.8 (15)
N9—C8—C7	131.96 (17)	Ag1—N17—H17A	80.7 (16)
N9—C8—C3	105.46 (15)	C16—N17—H17B	113.6 (17)
C7—C8—C3	122.57 (18)	Ag1—N17—H17B	128.7 (17)
C10—N9—C8	107.99 (15)	H17A—N17—H17B	114 (2)
C10—N9—H9	122.6 (19)	O19^ii^—N18—O19	122.1 (2)
C8—N9—H9	129.4 (19)	O19^ii^—N18—O20	118.96 (11)
N2—C10—N9	111.53 (15)	O19—N18—O20	118.96 (11)
N2—C10—C11	126.15 (15)		

Symmetry codes: (i) −*x*, −*y*, −*z*+1; (ii) −*x*, *y*, −*z*+1/2.

### Hydrogen-bond geometry (Å, º)

**Table:**

*D*—H···*A*	*D*—H	H···*A*	*D*···*A*	*D*—H···*A*
N9—H9···O20	0.81 (3)	2.05 (3)	2.8588 (18)	178 (3)
N17—H17*B*···O19^iii^	0.89 (3)	2.35 (3)	3.214 (2)	164 (2)

Symmetry code: (iii) −*x*, *y*−1, −*z*+1/2.
